# Targeting Neuroblastoma Stem Cells with Retinoic Acid and Proteasome Inhibitor

**DOI:** 10.1371/journal.pone.0076761

**Published:** 2013-10-07

**Authors:** Barbara Hämmerle, Yania Yañez, Sarai Palanca, Adela Cañete, Deborah J. Burks, Victoria Castel, Jaime Font de Mora

**Affiliations:** 1 Laboratory of Molecular Endocrinology, Centro de Investigación Príncipe Felipe, Valencia, Spain; 2 Paediatric Oncology Unit, Hospital La Fe, Valencia, Spain; 3 Laboratorio de Biología Molecular Hospital La Fe, Valencia, Spain; 4 Centro de Investigación Biomédica en Red de Diabetes y Enfermedades Metabólicas Asociadas (CIBERDEM), Instituto de Salud Carlos III, Barcelona, Spain; 5 Instituto Valenciano de Patología, Facultad de Medicina, Universidad Católica de Valencia San Vicente Mártir, Valencia, Spain; 6 Laboratory of Cellular and Molecular Biology, Fundación para la Investigación Hospital La Fe, Valencia, Spain; University of Colorado, School of Medicine, United States of America

## Abstract

**Background:**

Neuroblastma cell lines contain a side-population of cells which express stemness markers. These stem-like cells may represent the potential underlying mechanism for resistance to conventional therapy and recurrence of neuroblastoma in patients.

**Methodology/Principal Findings:**

To develop novel strategies for targeting the side-population of neurobastomas, we analyzed the effects of 13-cis-retinoic acid (RA) combined with the proteasome inhibitor MG132. The short-term action of the treatment was compared with effects after a 5-day recovery period during which both chemicals were withdrawn. RA induced growth arrest and differentiation of SH-SY5Y and SK-N-BE(2) neuroblastoma cell lines. Inhibition of the proteasome caused apoptosis in both cell lines, thus, revealing the critical role of this pathway in the regulated degradation of proteins involved in neuroblastoma proliferation and survival. The combination of RA with MG132 induced apoptosis in a dose-dependent manner, in addition to promoting G2/M arrest in treated cultures. Interestingly, expression of stem cell markers such as Nestin, Sox2, and Oct4 were reduced after the recovery period of combined treatment as compared with untreated cells or treated cells with either compound alone. Consistent with this, neurosphere formation was significantly impaired by the combined treatment of RA and MG132.

**Conclusions:**

Given that stem-like cells are associated with resistant to conventional therapy and are thought to be responsible for relapse, our results suggest that dual therapy of RA and proteasome inhibitor might be beneficial for targeting the side-population of cells associated residual disease in high-risk neuroblastoma.

## Introduction

Neuroblastoma is the most frequent extra-cranial solid tumor in children and high-risk cases still face poor prognosis due to therapy-resistant relapse [1,2]. To control minimal residual disease, high risk neuroblastoma is currently treated with the differentiating agent 13-cis-retinoic acid (RA) at completion of cytotoxic therapy [3,4]. Although this improves survival by 35% in children with metastatic neuroblastoma [4], the 5-year event-free survival rate still remains below 50%. Therefore, it is imperative to develop more effective therapeutic strategies to further improve long-term survival of patients.

Recent reports have shown that cellular response to RA can be increased by inhibiting proteasome-mediated RARα degradation which thereby increases RARα transcriptional activity. This further promotes retinoic acid-induced differentiation in both acute myeloid leukemia cells [5] and neuroblastoma cells [6]. Additionally, the ubiquitin-proteasome pathway regulates the activity of a variety of proteins that play crucial roles in tumor growth (p53, nuclear factor-κB (NF-κB), p27Kip1 among others). Bortezomib, a potent and selective inhibitor of the 26S proteasome, has already received approval by the Food and Drug Administration (FDA) for the treatment of relapsed or refractory multiple myeloma [7] and is currently being evaluated for the treatment of various cancers [8]. The activity of botezomib in neuroblstoma cells has also been explored, demonstrating its efficacy as an inhibitor of neuroblastoma cell growth [9]. However, some neuroblastoma cell lines display resistance to bortezomib through the activation of p38 MAPK [10]. Other mechanisms of bortezomib resistance are caused by point mutations in the critical domain for it’s binding [11] and in hypoxia-selected stem cells [12]. Therefore, a combination of therapies may be an effective strategy for circumventing development of bortezomib resistance.

It has been hypothesized that tumor-initiating cells that exhibit stem cell-like properties may be responsible for the failure of long-term remission of many cancers [13]. Thus, the major interest in targeting these side-population cells which express stemness markers is that they are highly tumorigenic and resistant to chemotherapy. Previous studies of neuroblastomas have identified a population of stem-like cells resistant to conventional therapeutic approaches [14]. With the present study, we have evaluated the effects of combining RA with proteasome inhibition on the growth and differentiation of stem-like cells of neuroblastoma lines. Our results provide evidence that this combination treatment targets neuroblastoma stem cells, restricting their proliferation for a prolonged period even after withdrawn of the compounds from the media. Thus, we have identified a combination of agents that may be beneficial for controlling recurrence of neuroblastoma in patients.

## Results

### Combined treatment with RA and the proteasome inhibitor MG132 attenuates neuroblastoma cell proliferation and induces apoptosis

**Figure 1:**
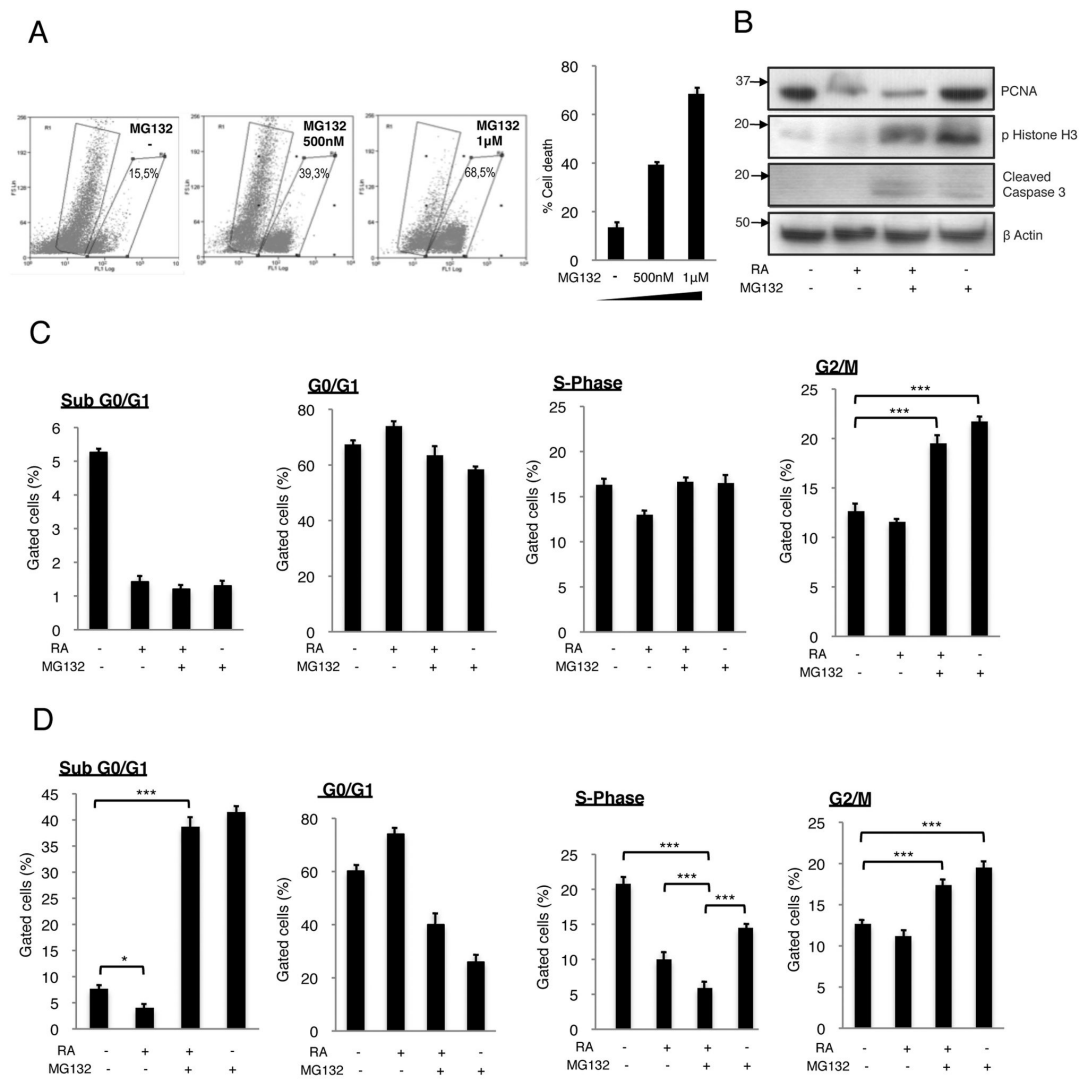
Effects of the combined RA/MG132 treatment on apoptosis and cell cycle. (**A**) The neuroblastoma cell line SK-N-BE(2) was treated with increasing doses of MG132 (100nM -1μM) for 3 days and analysed by flow cytometry using the fluorescent dye Yoyo1. Flow cytometry diagram and quantitative data. The percentage of cells is indicated in each quadrant. (**B**) Western blot analysis of the 4 treatment conditions after 3 days. (**C**) Quantitative data of the different phases of the cell cycle after 24h treatment. Notice that after 24h almost no apoptosis has been detected in MG132 and RA/MG132 cells. The increases of the percentage of cells in G2/M in response to combined treatment or to MG132 are statistically significant (asterisks). (**D**) Quantitative data of the different phases of the cell cycle after 72h of treatment.

To establish the working concentration for MG132, we initially treated the neuroblastoma cell line SK-N-BE(2) for 3 days with increasing concentrations of MG132 (ranging from 100nm to 1μM). The samples were subsequently analyzed by Western blot and flow cytometry using the dimeric cyanine nucleic acid dye Yoyo1. Consistent with previous reports on other neuroblastoma cell lines [10,15,16], we found that MG132 induces apoptosis in SK-N-BE(2) cells in a dose-dependent manner (Figure 1A). The effect of MG132 was similar in SH-SY5Y cells (unpublished data). Unless otherwise indicated, MG132 was used at 500nM in our experiments.

**Figure 2:**
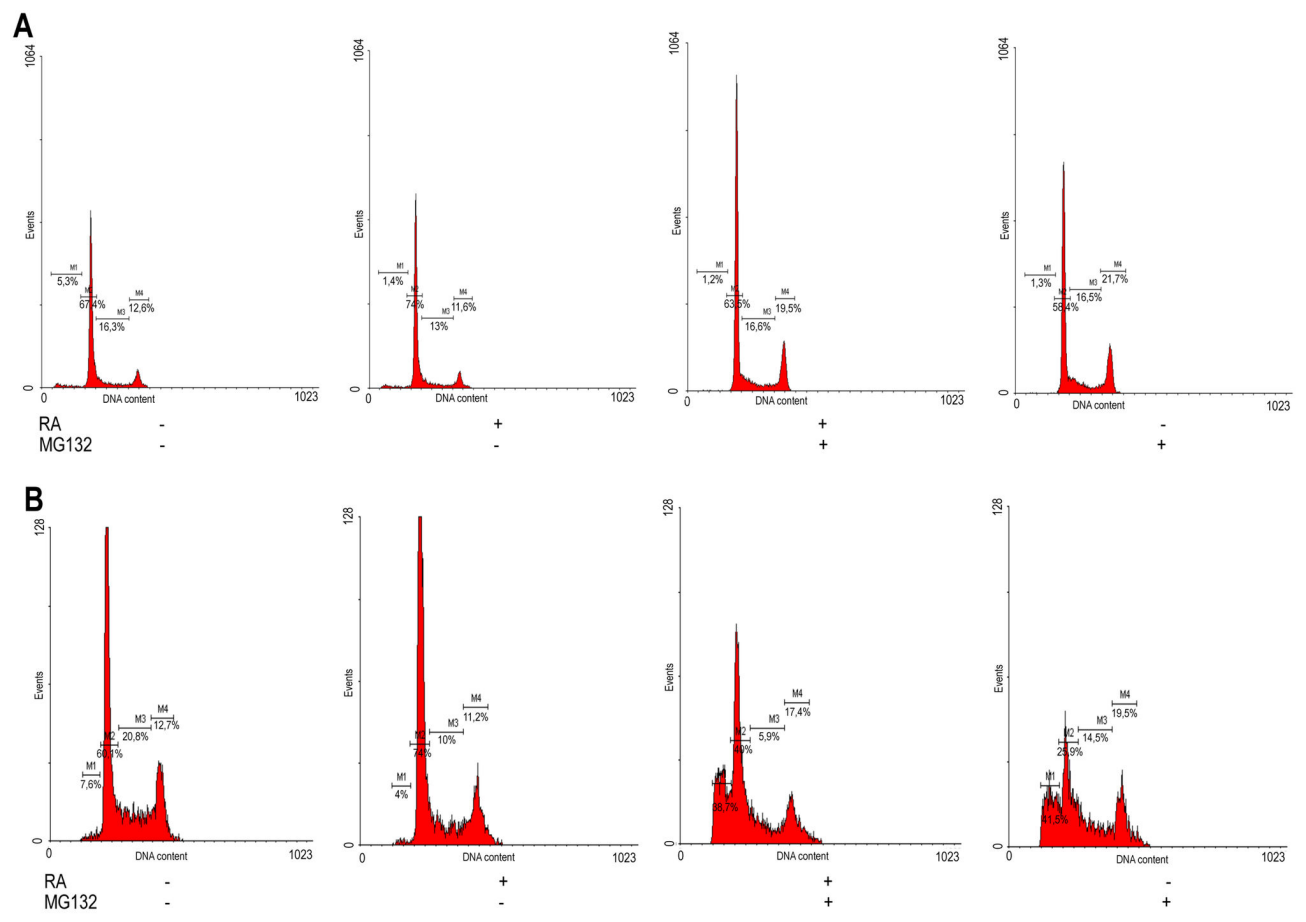
Effects of the combined RA/MG132 treatment on cell cycle (A) Flow cytometry diagram of propidium iodide stained cells after 24h treatment with RA and MG132. The percentage of cells is indicated in each quadrant. (**B**) Flow cytometry diagram of propidium iodide stained cells after 72h treatment with RA and MG132. The quantitative data of this experiment are shown in Figure 1C and D.

Treatment with RA alone reduced basal apoptosis in SK-N-BE(2) cells (Figure 1C, D and Figure 2). However, when RA was combined with MG132 for 3 days, the apoptosis rate was around 40%, only slightly lower than cells treated with MG132 alone (Figure 1B and 1D). Hence, the combined treatment counteracted the effects of RA on survival. MG132 reduced RA-dependent decrease of cells at S-phase after 3 days of treatment (compare both treatments with MG132 or with untreated cells in Figure 1D). PCNA expression diminished in response to RA alone or the combined RA/MG132 treatment (Figure 1B). These results suggest that the apoptotic effect of MG132 and the anti-proliferative effect of RA may be beneficial when combined together in therapy. A marked accumulation of cells at G2/M phase was detected after 24 hours of combined treatment and this effect persisted for 3 days when compared to RA alone or untreated cells (Figure 1C and 1D, see also Figure 2). Furthermore, we detected a significant increase of the mitotic marker phospho-histone H3 in cells treated with RA/MG132 and those treated with MG132 alone (see Figure 1B), suggesting that cells were blocked in mitosis rather than in G2 phase. Inhibition of the proteasome has been reported to induce G2/M cell cycle arrest in diverse cancer types, including neuroblastoma [17-22]. Our results indicate that the combination of RA with MG132 slows neuroblastoma growth by inducing apoptosis and by impairing specific phases of the cell cycle (mainly G2/M phase for MG132 and S phase for RA).

### The apoptosis induced by proteasome inhibition is independent of p53

**Figure 3:**
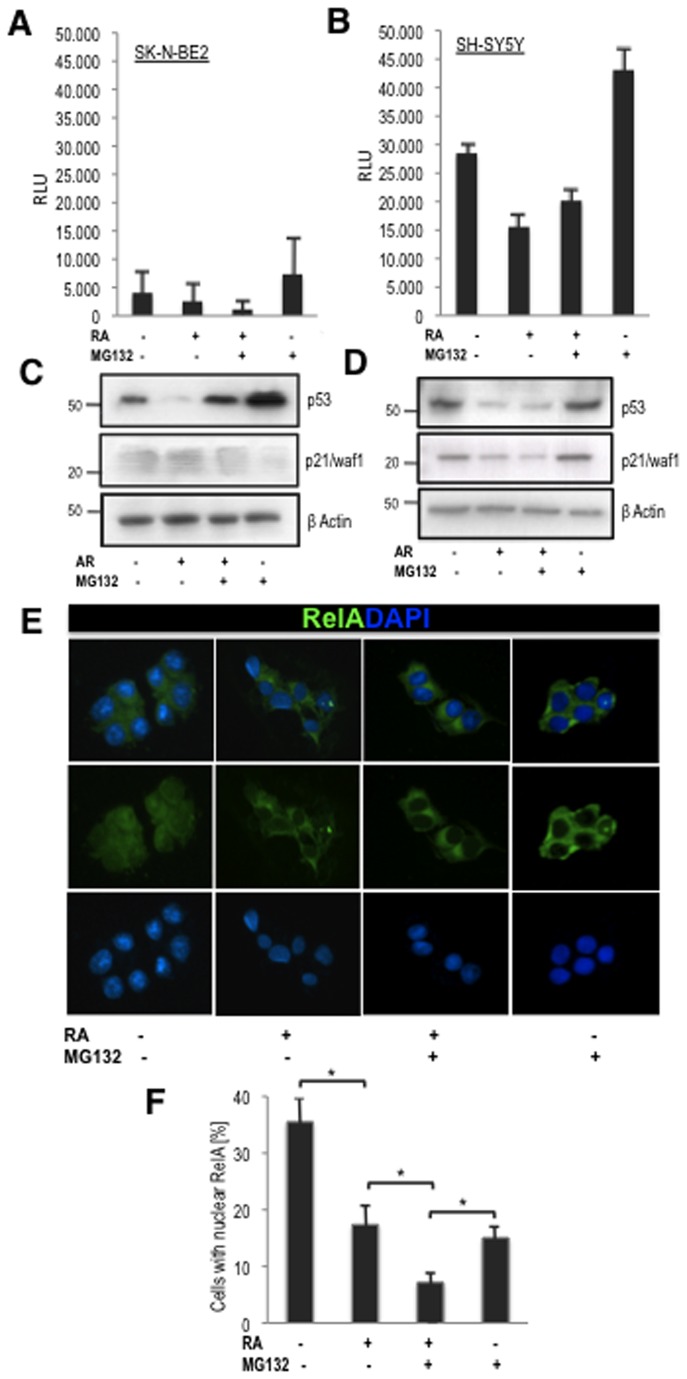
Combination of MG132 and RA induces p53-independent apoptosis and RelA inhibition. (A, B) The apoptosis induced by MG132 and MG132/RA is p53 independent. (**A**) Luciferase assay performed on SK-N-BE(2) cells and (**B**) SH-SY5Y cells to assess transcriptional activity of p53 upon treatment. SK-N-BE(2) cells express mutant p53, SH-SY5Y neuroblastoma cells are wildtype for p53. LRU= Luciferase reference unit. (**C**) Representative western blot showing the accumulation of p53 protein in response to MG132 treatment. MG132 treatment does not induce up-regulation of the p53 responsive gene p21/Waf1 in SK-N-BE(2) cells, (**D**) in contrast to SH-SY5Y cells. (**E**, **F**) Effects of combined RA/MG132 treatment on NF-κB signalling. Both MG132 as well as RA cause significant decrease of nuclear expression of RelA (p65) protein. (**E**) Immunofluorescent detection of sub-cellular localization of RelA (p65) protein under the different treatment conditions. Arrows indicate RelA (p65) protein in the nucleus. Scale bar = 25μm. (**F**) Quantitative data. The reduction of the cell fraction with nuclear RelA expression in response to combined treatment is statistically significant (*) as compared to control (p< 0.037), to RA treated cells (*p*=0.037), but also to MG132 treated cells (*p*=0.016).

Inhibition of the proteasome stabilizes, among other proteins, the tumor suppressor p53 [23]. Thus, we observed an accumulation of p53 protein in the SK-N-BE(2) neuroblastoma cells in response to proteasome inhibition (Figure 3C). The SK-N-BE(2) cell line expresses mutant p53 [24] which would not be expected to respond to proteasome inhibition. Luciferase assays confirmed that the effects of MG132 were independent of p53 transcriptional activity in SK-N-BE(2) cells but not in SH-SY5Y cells where p53 is not altered, suggesting that response to this inhibitor may be cell-type dependent (Figure 3A and B). Consistent with this, p53-regulated genes such as p21/Waf1 (Figure 3C) were not altered in the SK-N-BE(2) cell line.

### The combination of RA and MG132 interferes with NF-κB nuclear localization

The transcription factor NF-κB (nuclear factor kappa B) has emerged as a crucial regulator of cell survival [25,26], and several studies have provided compelling evidence that aberrant NF-κB activation contributes to inappropriate cell survival in a wide range of solid and hematopoietic malignances [27,28]. Interestingly, proteasome inhibition interferes with the activation of NF-κB [29-31]. Thus, we assessed whether the combination of MG132 and RA modulates NF-κB signalling in neuroblastoma cells by quantifying nuclear anti-RelA antibody staining for each treatment condition. Distinct nuclear localization of RelA (p65) was observed in 35% of untreated cells; whereas this was reduced to around 15% in cells treated with either RA or MG132 alone. When cells were treated with both chemicals combined, nuclear RelA (p65) was detected in only about 7% of the culture (Figure 3F and G). Thus, the combination of MG132 and RA displayed synergistic inhibition of NF-κB nuclear translocation.

### Combined RA/MG132 treatment reduces long-term expression of stem cell-related genes

**Figure 4:**
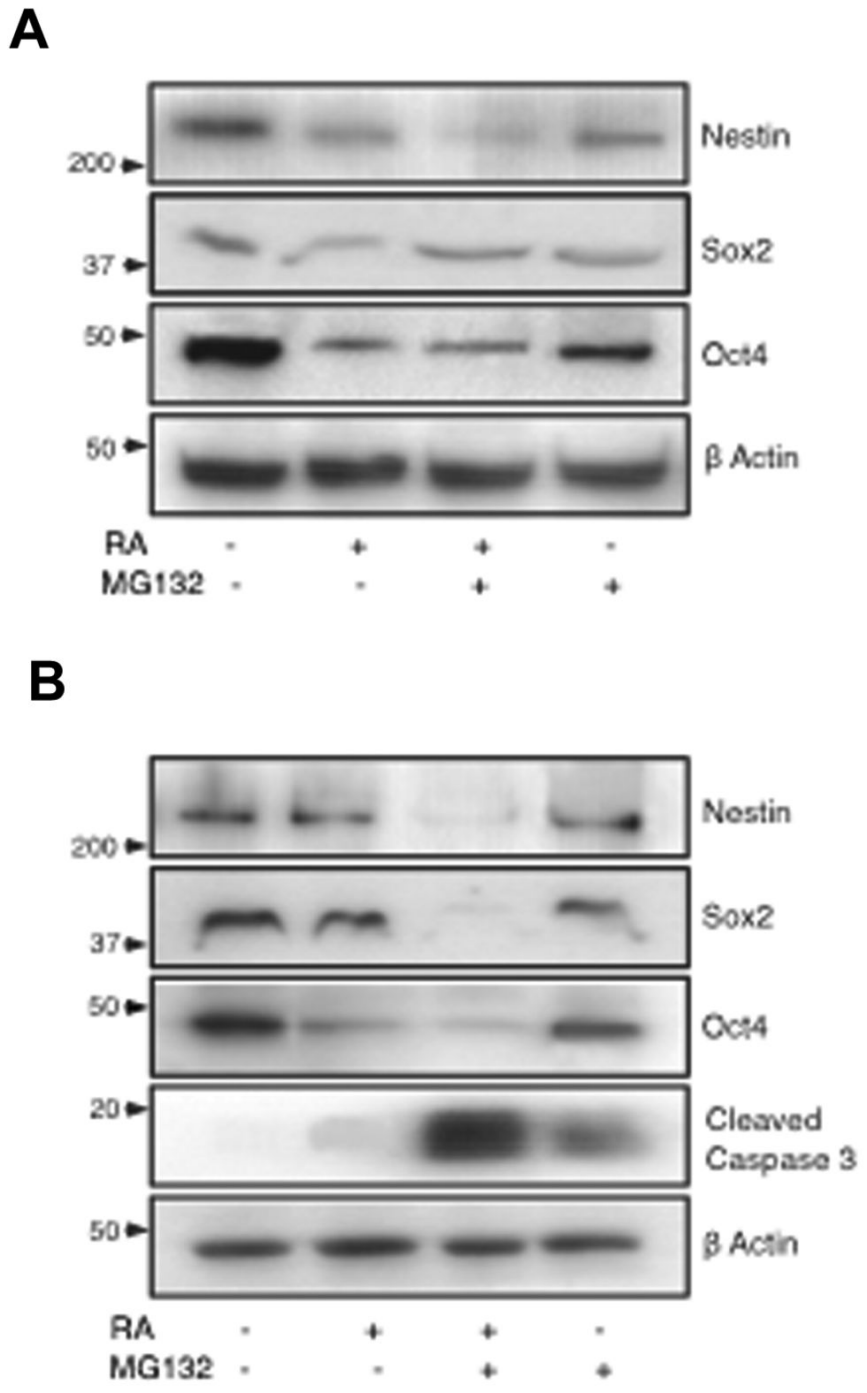
Effects of the combined RA/MG132 treatment on the expression stem cell- related markers. (**A**) Western blot analysis at completion of a 3 day treatment with (+) or without (-) RA and/or MG132, as indicated, and (**B**) at completion of a 3 day treatment plus 5 additional days in the absence of compounds.

**Figure 5:**
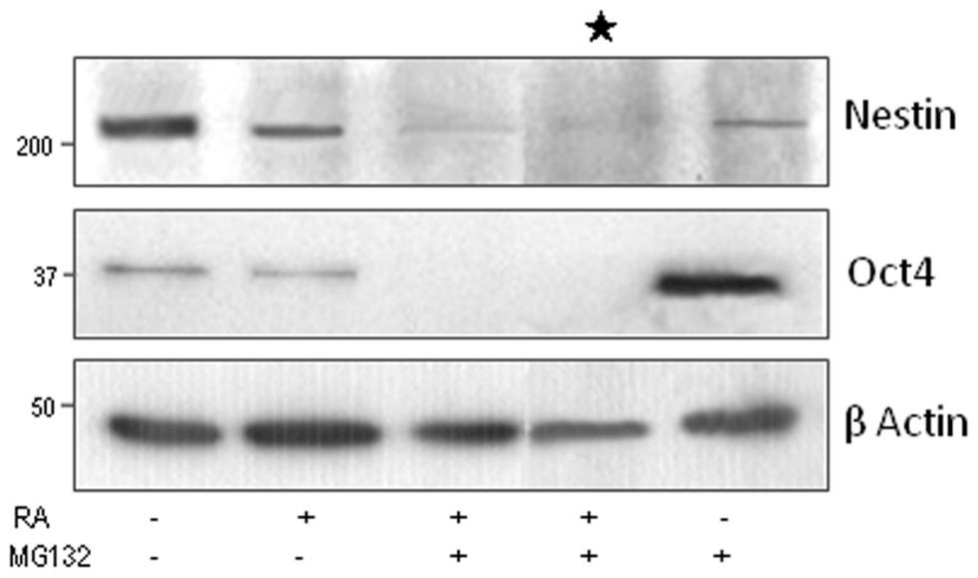
Combination of MG132 and RA reduces long-term expression of stemness markers. Western blot analysis at completion of a 3 day treatment with (+) or without (-) RA and/or MG132, as indicated, plus 5 additional days in the absence of compounds. In the lane labelled with an asterisk, the recovery was performed with conditioned medium from untreated cells.

**Figure 6:**
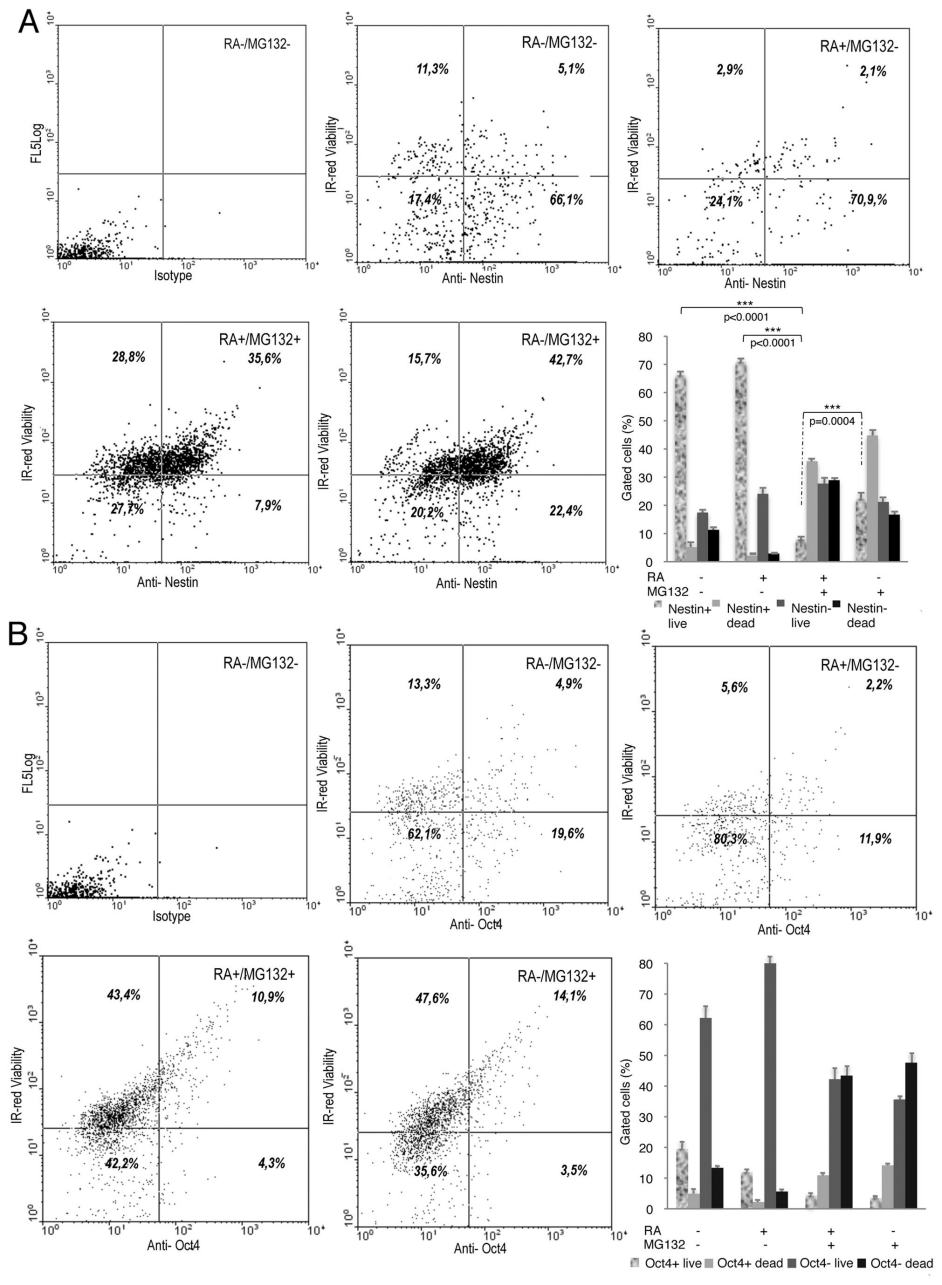
Effects of the combined RA/MG132 treatment on the expression of Nestin, Oct4 and IR-red viability at completion of a 3-day treatment. (**A**) Cells were treated with (+) or without (-) RA and/or MG132, as indicated, for 3 days. Flow cytometry dot plots and graphical representation of viable Nestin labelled (+) or not labelled (-) cells. The percentage of cells is indicated in each quadrant. (**B**) Flow cytometry dot plots and graphical representation of viable Oct4 labelled (+) or not labelled (-) cells. The percentage of cells is indicated in each quadrant.

**Figure 7:**
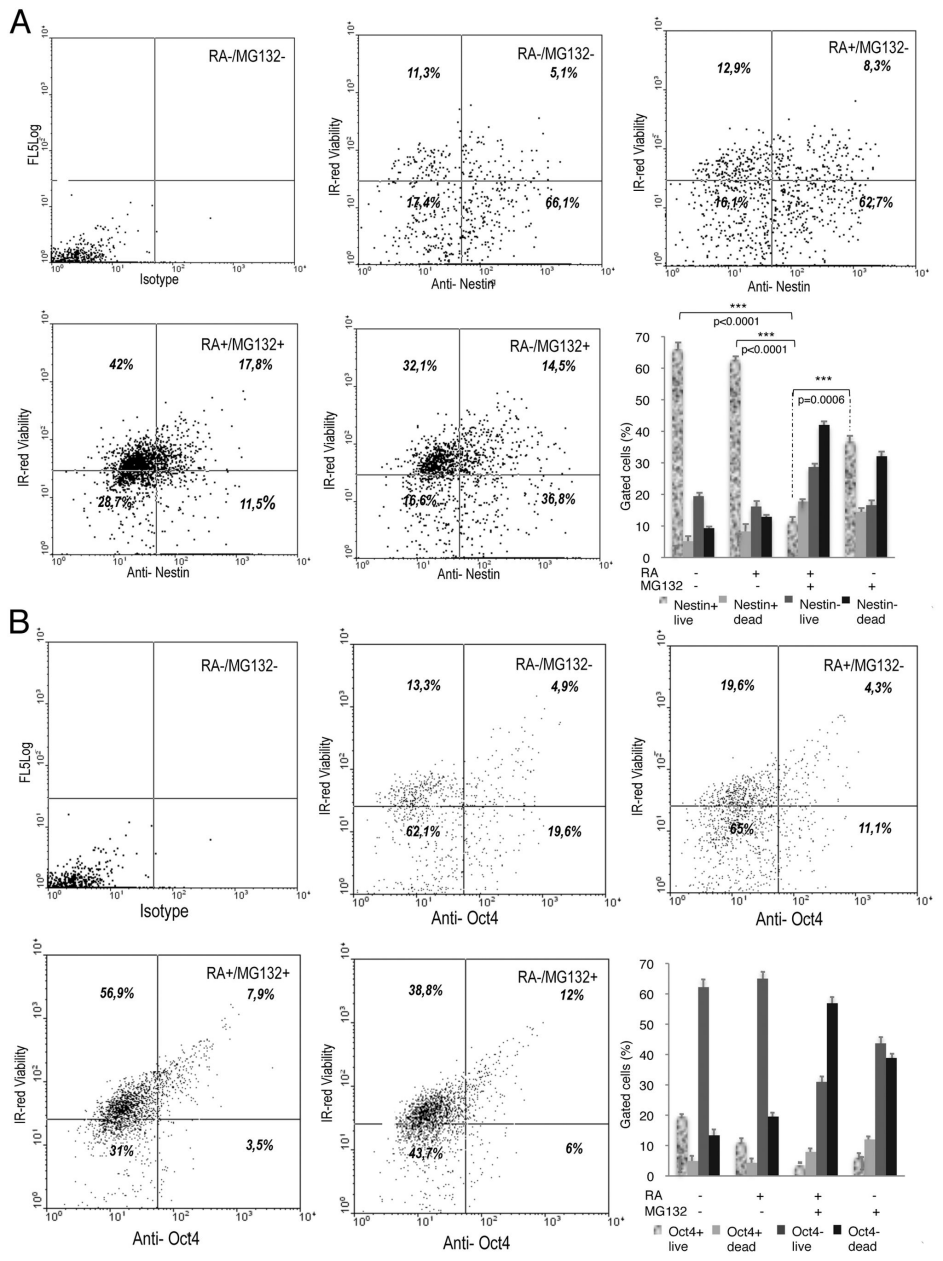
Effects of the combined RA/MG132 treatment on the expression of Nestin, Oct4 and IR-red viability at completion of a 3 day treatment plus 5 additional days of recovery without compounds. (**A**) Flow cytometry dot plots and graphical representation of viable Nestin labelled (+) or not labelled (-) cells. The percentage of cells is indicated in each quadrant. (**B**) Flow cytometry dot plots and graphical representation of viable Oct4 labelled (+) or not labelled (-) cells. The percentage of cells is indicated in each quadrant.

To evaluate the potential usefulness of these chemicals for targeting the stem-like cells of neuroblastomas, we focused on SK-N-BE(2) cells due to their reported malignant potential including the amplification of MYCN expression and presence of a verapamil-sensitive side population [14]. Cells were analysed for the expression of the stemness markers Oct4, Sox2 and Nanog as well as the neural progenitor markers CD34 and Nestin. Treatment with RA/MG132 for 3 days decreased protein expression levels of Sox2, Oct4, and Nestin (Figure 4A). Surprisingly, the levels of these proteins remained very low 5 days after the combination of chemicals was withdrawn from the cell culture medium, suggestive of a long-term effect on the population of stem-like cells (Figure 4B). These observations are consistent with the persistent apoptosis detected in the post-treatment phase of cells which received RA/MG132 (Figure 1) and with the enhanced levels of cleaved-caspase 3 (Figure 4B), long-term loss of expression of stemness markers (Figure 5) and by the high percentage of dead cells detected by flow cytometry (Figures 6 and 7).

**Figure 8:**
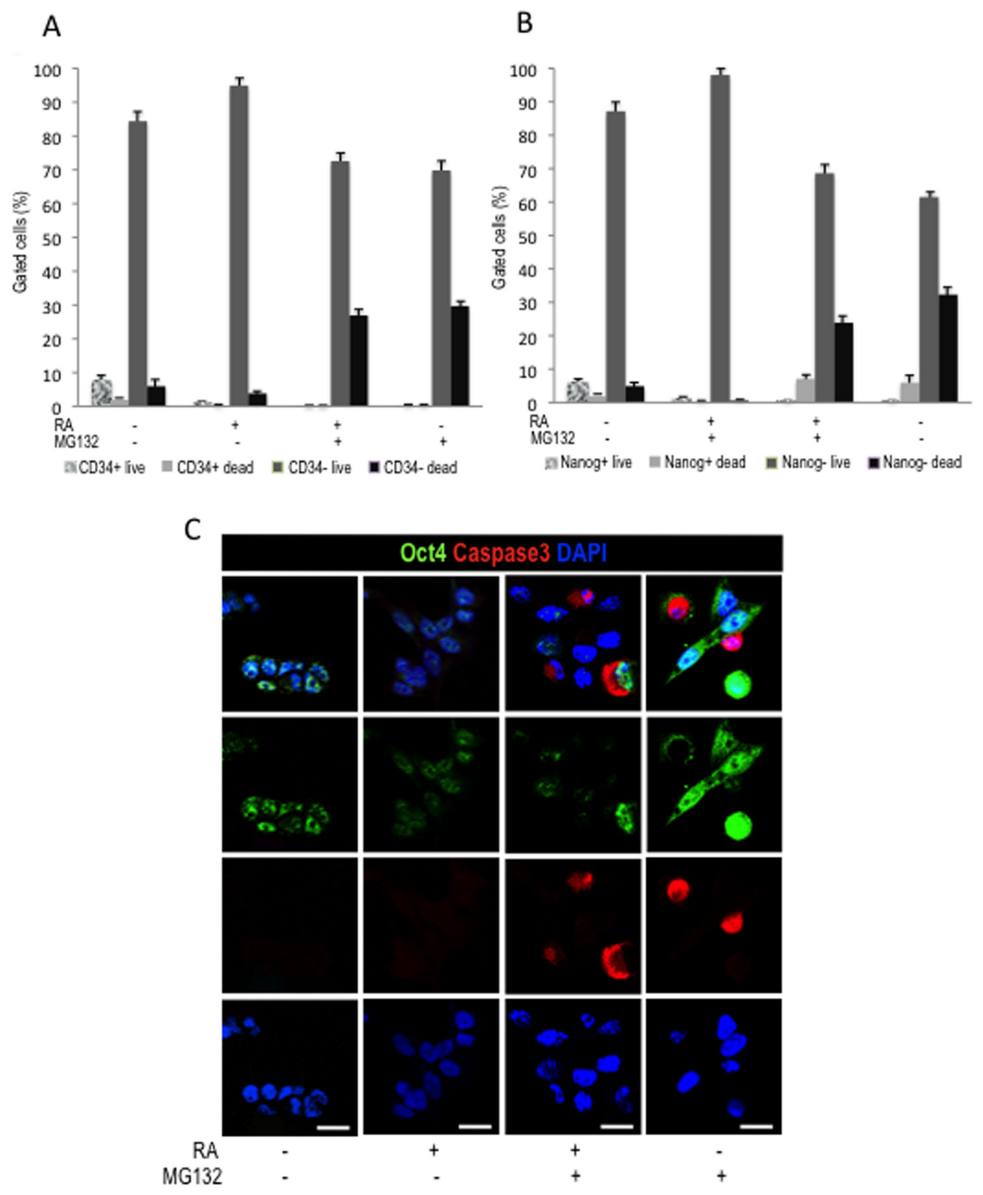
Study of viability and expression of stem-cell markers of cells treated with MG132 and RA. (**A**) Quantitative data of CD34-expressing cells (+) or non-expressing cells (-) that are dead or remain alive after the treatment at completion of a 3 day treatment. (**B**) Quantification of Nanog-expressing (+) or non-expressing cells (-) at completion of a 3 day treatment. (**C**) Confocal microscopy analysis of Oct4 stained cells at completion of a 3 day treatment. Scale bar = 15μm.

To determine whether this marked cell death was related with secretion of self-renewing growth factors, or alternatively to an inherent mechanism already activated in cells, cultures were maintained in conditioned medium after drug withdrawal. However, even under these conditions, apoptosis persisted and the expression levels of Oct4 and Nestin were comparable to cells which were grown in normal medium after drug removal (Figure 5). Flow cytometry revealed a reduction of Nestin-expressing live cells after completion of combined treatment (Figure 6A). Expression of Oct4, CD34 and Nanog was also diminished in live cells after the combined treatment (Figures 6B and 8). However, no differences in the expression levels of these three markers were observed among the different treatments. Interestingly, 5 days after treatment withdrawal, a significant decrease of live cells expressing Oct4 (Figure 7B) persisted in cultures treated with RA together with MG132. By contrast, cultures treated with MG132 alone showed increased percentage of Oct4^+^ living cells (3.5%±0.5 immediately after treatment vs. 6±0.9 5 days after treatment cessation, *p*=0.0065) (Figure 7B). After the recovery period, the proportion of Nestin-expressing live cells previously treated with the combination of RA and MG132 was substantially lower than in untreated cells or treated with only one of the compounds (Figure 7A). It is surprising, however, that the percentage of Oct4^+^ dead cells was slightly higher in cells treated with MG132 alone than in those that received combined treatment (14.15%±1.2 vs. 10.9±0.9, respectively, *p*=0.0287), and 5 days after treatment withdrawal (12%±1.1 vs. 8.2±0.5, respectively, *p*=0.0047) (graphics in Figures 6B and 7B). The apparent reduction of apoptosis in Oct4-expressing cells achieved with the combined treatment vs. MG132 alone contrasts with the reduction in both, overall Oct4 expression (Figure 4) and the percentage of cells expressing Oct4 (graphic in Figure 6B). This result suggests that apoptosis might not be the only explanation for the decreased proportion of live cells expressing stem cell-related marker proteins in response to combined treatment. Thus, RA/MG132-induced differentiation may additionally alter the population expressing stem-cell markers.

### The combination of RA and MG132 induces differentiation of surviving cells and reduces neurosphere formation capacity

**Figure 9:**
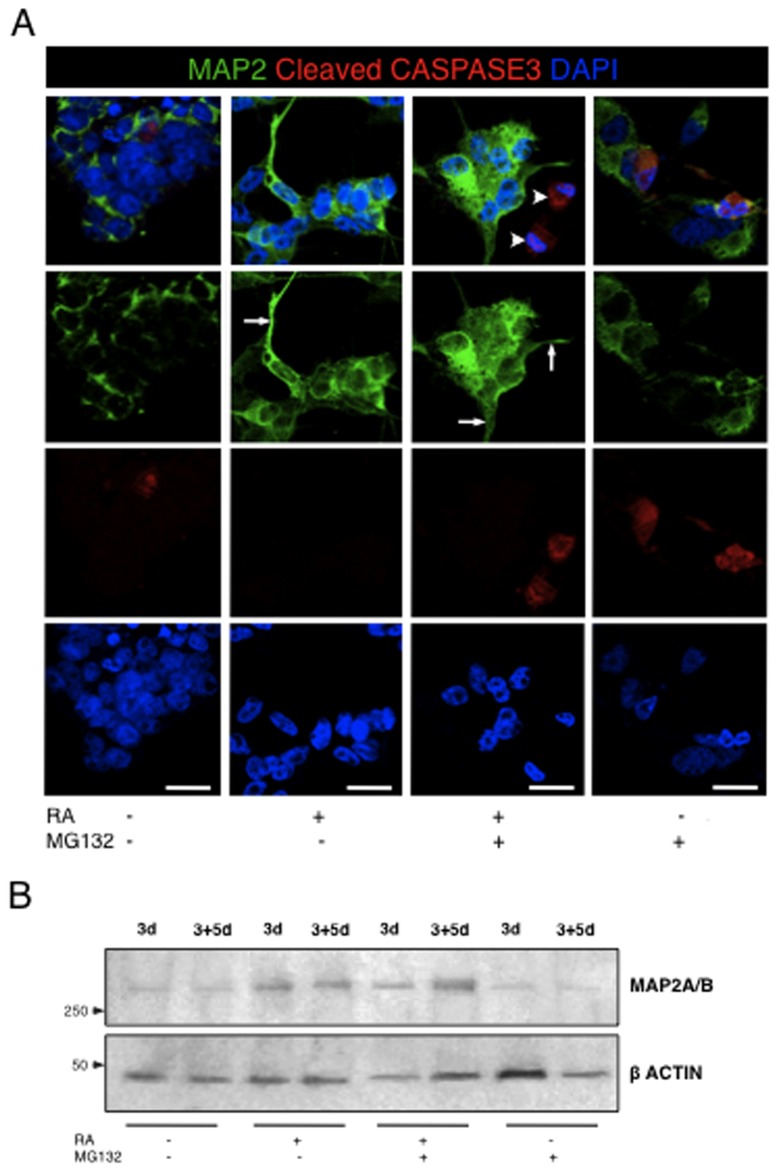
Effects of the combined RA/MG132 treatment on neuronal differentiation. (**A**) Cells were treated with (+) or without (-) RA and/or MG132, as indicated. Confocal microscopy analysis of MAP2 stained cells at completion of a 3 day treatment. Scale bar = 20µm. (**B**) Western blot analysis of MAP2 at completion of a 3 day treatment and after a recovery period of 5 additional days.

Recent reports have suggested that combination of proteasome inhibition and retinoic acid potentiates differentiation in acute myloid leukemia cells [5], and in SH-SY5Y neuroblastoma cells [6]. Hence, we tested whether the combination of RA and MG132 would also induce differentiation in SK-N-BE(2) neuroblastoma cells. We used a low MG132 concentration (100nM) to ensure that the apoptosis rate would not exceed 20% during the differentiation protocol. Cells were treated during 3 days, fixed and immunostained with an antibody against MAP2 (microtubule-associated protein), a neuron-specific cytoskeletal protein that is enriched in dendrites, along with an antibody against cleaved caspase 3 (Figure 9A).

**Figure 10:**
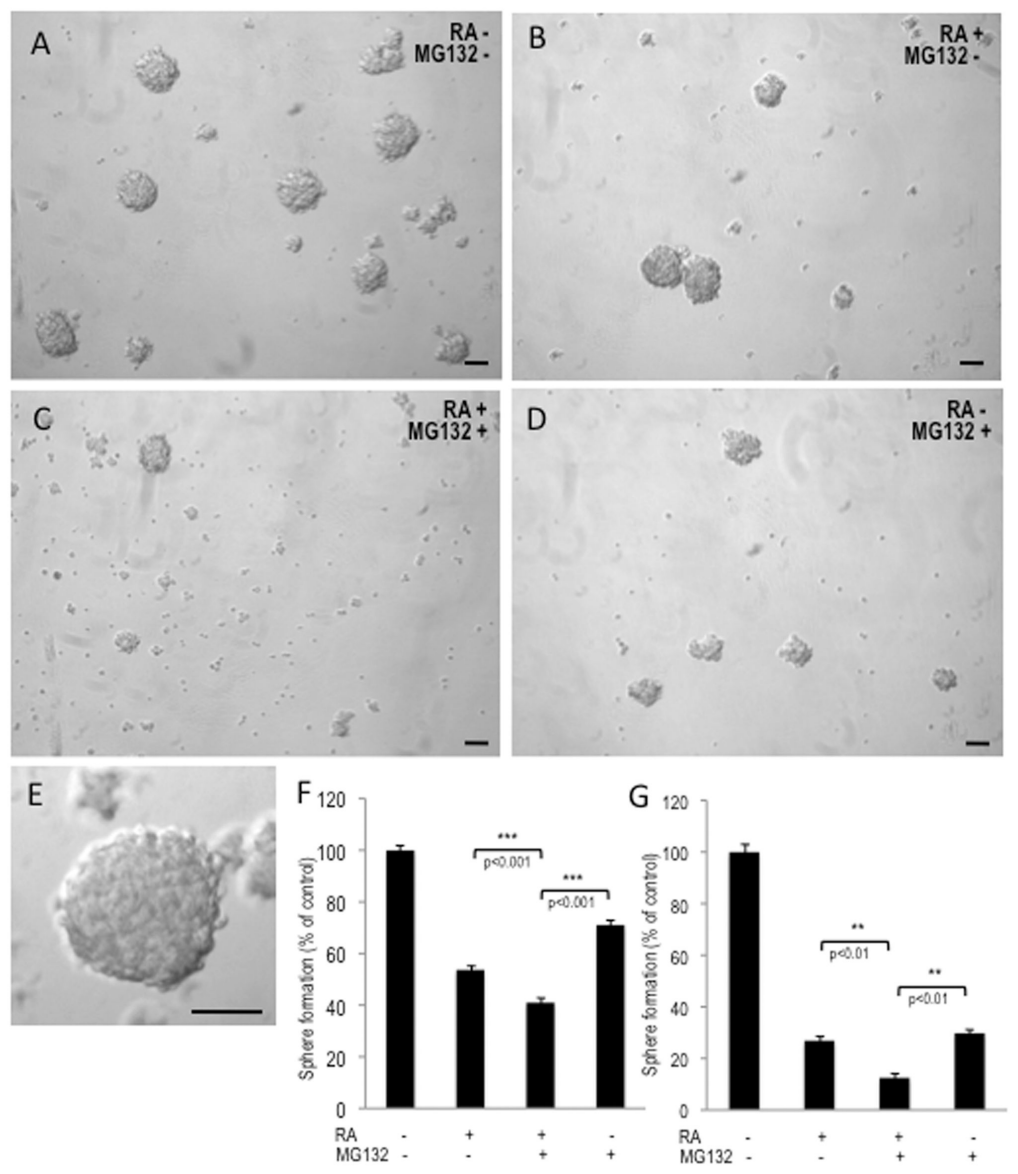
Combined treatment with RA and MG132 impairs neurosphere formation. (**A**-**D**) After completion of a 3-day treatment, SK-N-BE(2) cells were seeded on an ultra-low attachment 6-well plates and cultured in sphere medium (SM). After 9 days of culture, cells were examined by phase-contrast microscopy. Scale bars: 50µM. (**E**) Higher magnification of a sphere, scale bar: 50µM. (**F**) Neurospheres ≥50 µm were quantified after five days in SM culture and (**G**) the same cultures were quantified after 9 days in SM culture, defined as the percentage of spheres in each sample in relation to that in a control sample (without RA and MG132). The primary assay in (F) shows the percentage of stem/progenitor cells in the population, and the secondary assay in (G) shows self-renewal capability. Data represents means ± SE of three independent experiments.

Cells treated with RA alone or in combination with MG132 stained prominently for MAP2 staining and displayed the differentiated morphology of neurite development (arrows). Interestingly, most apoptotic cells of the RA/MG132-treated cultures did not express MAP2 (arrowheads). Western blot analysis confirmed increased expression of MAP2 protein in both RA and RA/MG132 treated samples; up-regulation of MAP2 was equivalent between cultures that were analysed immediately after treatment or after the recovery period of 5 days (Figure 9B). Thus, our results suggest that the combined RA/MG132 treatment induces neuronal differentiation in cells of the SK-N-BE(2) neuroblastoma line that escape apoptosis. Moreover, the combination of proteasome inhibition and RA reduces the number of live cells expressing stem cell-related markers, even 5-days after cessation of treatment. To further confirm the synergistic effects of RA and MG132 in reducing the stem-like population of this neuroblastoma line, we performed a sphere formation assay. Treatment with RA/MG132 was more potent at inhibiting sphere formation than either compound alone (Figure 10).

## Discussion

Therapy for high-risk patients includes the differentiating agent RA; however more than 50% of patients relapse, which could be due, at least in part, to the generation of resistance to retinoid [32]. Thus, more information about the effects of RA is needed to improve the retinoid component of therapy for patients with minimal residual disease and to develop synergistic combination therapies which include RA. In the present study, we analyzed the effects of RA combined with proteasome inhibition in SK-N-BE(2) neuroblastoma cells. This combined treatment caused apoptosis in a dose-dependent manner together with growth arrest and differentiation. The dose of the proteasome inhibitor MG132 (500nM) that we employed to obtain apoptosis rates of about 50% was relatively high when compared to other studies in which Bortezomib was used on neuroblastoma cell lines [10,15]. Peptide boronic acid proteasome inhibitors which include Bortezomib are more potent than their peptide aldehyde analogues such as MG132 [33].

A recent study [34] has suggested that the sensitivity to proteasome inhibition is associated with the status of p53. However, apoptosis triggered by proteasome inhibition appears to be independent of p53 in prostate cancer [35], multiple myeloma [30], and colon cancer cells [36]. Moreover, in breast and lung cancer [20,37], sensitivity to proteasome inhibition seems to be only partially dependent on p53. Therefore, the degree to which p53 status modulates sensitivity to proteasomal inhibition may be, in fact, cell-type dependent. The relationship between p53 and the proteasome in neuroblastomas also appears to vary depending on the cell line [10,15,16]. The SK-N-BE(2) cell line used in the present study was derived from a neuroblastoma patient after chemotherapy and contains a missense mutation which inactivates p53. Our data confirm that the apoptosis induced by MG132 and by the combination of RA/MG132 is independent of p53 in SK-N-BE(2) neuroblastoma cells.

Hagenbuchner et al. 2010 demonstrated that Bortezomib-induced apoptosis in neuroblastoma cells activates the pro-apoptotic BH3-only proteins Noxa and Puma and induces repression of the anti-apoptotic Bcl2 family member Bcl-xL. Thus, we assessed the pathways implicated in the apoptotic effects of the proteasome inhibitor MG132 when combined with RA. Proteasome inhibitors, such as Bortezomib or MG132, are well known NF-κB inhibitors [8,37]. Based on the sub-cellular localization of RelA (p65) proteins in our experiments, MG132 blocks NF-κB signalling when administered with RA to SK-N-BE(2) neuroblastoma cells. Some forms of retinoic acid produce a reduction in NF-κB activity in human malignant keratinocytes [38].

NF-κB regulates a variety of genes implicated in cell proliferation and cell survival. Therefore, in many different types of human tumors, including high risk neuroblastoma [39], NF-κB is constitutively active and drives cell proliferation. NF-kB is also linked to the immune regulation of neuroblastomas; low levels of NF-kB are associated with reduced expression of MHC-1 complexes. Overexpression of NF-kB p65 together with Interferon Regulatory Factor 1 was able to restore MHC-1 expression and cellular immune complex formation in neuroblastoma cell lines [40]. However, in intestinal cancer, NF-κB signalling enhances Wnt activation and induces de-differentiation of non-stem cells that acquire tumor-initiating capacity [41]. These observations suggest that NF-κB signalling may be a therapeutic target depending on the type of cancer.

Various lines of evidence suggest that stem-like cells are responsible for failure of long-term remission [42]. Thus, eradicating tumors may be difficult because conventional treatments target the bulk of tumor cells rather than these tumor-initiating stem cells which are chemoresistant. The side-population assay is widely used for the identification and isolation of stem-like cells from cancers based on their capacity to exclude dyes such as Hoechst 33342.

To determine whether the RA/MG132 combination alters the population of stem-like cells of neuroblastomas, we analysed the expression of stem cell-related markers such as Oct4, Nanog and Sox2 which are key regulators of embryonic stem cell maintenance [43] and are overexpressed in different cancers, including neuroblastoma [44]. The neural progenitor markers Nestin and CD34 are also expressed in neuroblastoma cells [14]. The exact role of these stem cell-related genes in tumors is not completely clear, but Nanog [45], Oct4 [46] and Nestin [47] have been associated with a more immature and aggressive cell phenotype. In our studies, protein levels of Oct4, Sox2, and Nanog were significantly reduced by RA/MG132 combined treatment. Remarkably, this reduction of stem cell markers persisted during the 5 days after treatment cessation. Moreover, the proportion of live cells expressing Nestin and Oct4 was significantly lower in cells receiving RA/MG132 than in control cultures or those treated with only one of the compounds. This effect was accompanied by persistent apoptosis and differentiation of those cells that escaped apoptosis, suggesting that RA/MG132 may be beneficial to prevent neuroblastoma relapse.

The standard treatment for high-risk neuroblastoma patients includes RA and immunotherapy with anti-GD2 antibodies. The objective of this approach is eradication of minimal residual disease which is present in over half of children who have achieved complete remission by imaging criteria. However, even with this intensive treatment, many children relapse and eventually die from disease progression. Our data reveal the novel observation that proteasome inhibition administered in combination with RA induces apoptosis in stem-cell like cells of neuroblastoma cell lines. The combined effects of RA/MG132 were more potent at reducing the stem-like cell population than either compound alone and moreover, impaired their capacity to form neurospheres. Therefore, we predict that this combined treatment might also have a positive impact *in vivo* in animal models. In human acute myeloid leukemia cells, bortezomib also sensitizes to RA-induced differentiation [48]. However, our results also show increased apoptosis, suggesting that the molecular targets between these two diseases might be different, such as the activation of the JNK pathway [6], or on whether bortezomib is given after or concomitantly with RA [49].

Since cancer stem cells are frequently resistant to conventional therapy and are responsible for relapse, our results suggest that dual therapy might be beneficial for improving the outcome of patients with high-risk neuroblastoma. RA is the current standard treatment in the control of minimal residual disease in high-risk neuroblastoma patients and Bortezomib is already approved by EMA/FDA. Development of therapies for pediatric cancers is complicated by the rarity of these diseases with respect to the total population and the fact that only a limited number of drugs can be tested. Hence, drug combination therapies, particularly with drugs that are already approved, may play a key role in future neuroblastoma treatment strategies.

## Materials and Methods

### Cell culture and treatments

SK-N-BE(2) and SK-SY5Y cell lines were purchased from ATCC. Both cell lines were maintained in DMEM-F12 medium containing 10% fetal bovine serum (FBS). Most experiments were carried out on SK-N-BE(2) cells. Cells were treated with 13-cis-retinoic acid (10 µM, Sigma) and/or with the proteasome inhibitor MG132 (500nM if not specified otherwise, Calbiochem). Untreated control cells were cultured in the presence of 0.1% DMSO. We used MG132, a synthetic analogue of the FDA-approved dipeptidyl boronic acid compound Bortezomib. Both, MG132 and Bortezomib are highly selective, reversible inhibitors of the 26S proteasome.

To establish the working concentration for MG132 we initially treated for 3 days the neuroblastoma cell line SK-N-BE(2) with increasing doses of MG132 (ranging from 100nm to 1μM). The samples were subsequently analysed by western blot and flow cytometry using the dimeric cyanine nucleic acid dye Yoyo1. In accordance with previous reports on neuroblastoma cell lines [10,15], we found that MG132 also induces apoptosis in SK-N-BE(2) cells in a dose dependent manner (Figure 1A). Thus, we administered MG132 at 500nM unless otherwise indicated, whereby apoptosis rate was around 40%. The concentration of RA, was 10µM in all experiments, since concentrations between 5-10 µM are well known to be effective against neuroblastoma in vitro (Reynolds et al, 1994). Moreover, these drug levels are equivalent to known pharmacological dosages used in clinical trials of RA administered orally to neuroblastoma patients [50,51].

### Antibodies

Monoclonal antibodies were used as follows: anti-p53 (clone DO-1), anti-PCNA (clone PC10) and anti-p21waf1 (clone F-5) from Santa Cruz Biotech; anti-Oct 4 (7F9.2, Millipore), anti-Nestin (BD611658, BD Transductions Laboratory ^TM^; anti-β-actin (clone ac-15) and anti-MAP2 (clone HM-2) from Sigma-Aldrich. In addition, the following polyclonal antibodies were used: anti-cleaved Caspase 3 (Cell signalling); anti-RelA (Santa Cruz Biotech); anti-Sox2 (R&D Systems), and anti-phoshohistone H3 from Cell Signaling.

### Western blotting

Conventional lysates were done with commercial lysis buffer (Cell Signaling) supplemented with protease cocktail inhibitors (Roche), up to 150 mmol/L NaCl, 1 mmol/L AEBSF (Roche), and 1 mmol/L NaF. Proteins were transferred to Immobilon-P membranes (Millipore) and blotted with the indicated antibodies.

### Flow cytometry analysis

For cell cycle analysis, cells were harvested by trypsin treatment, washed with PBS and resuspended in 70% ethanol to permit fixation and permeabilization at −20°C. Propidium iodide-stained cells (5 µg/ml; Sigma) were analysed using a flow cytometer (Cytomics FC500; Beckam Coulter). For the analysis of stem cell-related markers expression, cells were harvested by trypsin treatment, labelled with the fluorescent dye IR_red viability (Invitrogen, Fisher), fixed in 4% paraformaldehyd, permeabilized and stained for Oct4, Nestin or Nanog-PE. In the case of CD34, unfixed cells were stained with CD34-PE antibody along with the dimeric cyanine nucleic acid dye Yoyo1.

### Immunofluorescence

Cells were fixed with 4% paraformaldehyde for 15 minutes, permeabilized in PBS containing 0.1% Triton-X100 for 15 minutes, and blocked for 60 minutes, at room temperature with 1% horse serum and 3% BSA in PBS. Cells were incubated overnight with the primary antibodies indicated above. The secondary antibodies conjugated to Alexa 488, Alexa 568 or Alexa 591 (Invitrogen) were used as recommended by the supplier. Counterstaining of nuclei was performed with DAPI. The samples were imaged on a Leica TCS SP2 confocal microscope or on a Leica DM6000 fluorescence microscope.

### Luciferase Assay and vectors

Cells were transfected with the p53 RE-luciferase vector using Lipofectamine 2000. All luciferase assays were performed with Luciferase Assay System (Promega, Madison, WI), and were conducted essentially according to the manufacturer’s protocol. The measurements were carried out with a luminometer (Berthold).

### Statistical Analysis

Experiments were carried out in triplicates in at least two independent experiments, and data were expressed as the mean ±SE. Statistical significance was assessed by Student’s two-tailed unpaired t-test. P < 0.05 was considered statistically significant. All statistical analyses were performed using InStat 3.1a software (GraphPad Scientific Software, Inc, La Jolla, CA).

### Neurosphere formation assay

SK-N- BE(2) cells were treated as indicated and 3 days later cells were seeded at a density of 2x10^4^ cells/well in 6-well ultra-low attachment culture plates. Sphere-promoting medium (SM) contained a 50:50 mix of F12 and DMEM (Invitrogen, Fisher Scientific), supplemented with 20 ng/ml EGF, 40ng/ml bFGF, 1% B27 supplement (Invitrogen, Fisher Scientific), 0.1 mM β-mercaptoethanol (Sigma) and 100 units/ml penicillin/streptomycin (Invitrogen, Fisher Scientific). The medium was changed every 4-5 days and neurospheres were mechanically dissociated. On day 15, cultures were examined with a Leica DM6000 phase contrast- microscope for neurite formation.
